# The Predictive Power of Early Socio-Emotional Skills on Behavioral Outcomes in Very Preterm Preschoolers: A Longitudinal Study

**DOI:** 10.3390/pediatric18020060

**Published:** 2026-04-20

**Authors:** Chiara Ionio, Caterina Colombo, Francesco Cavigioli, Francesca Sala, Rachele Cantella, Marina Balestriero, Giovanna Cardile, Giulia Ciuffo, Gianluca Lista

**Affiliations:** 1 CRIdee, Unità di Ricerca sul Trauma, Dipartimento di Psicologia, Facoltà di Psicologia, Università Cattolica del Sacro Cuore, 20123 Milan, Italy; 2 Neonatologia Patologia e Terapia Intensiva Neonatale, Ospedale dei Bambini “Vittore Buzzi”, ASST Fatebenefratelli Sacco, Via Castelvetro 32, 20154 Milano, Italy; 3 Facoltà di Psicologia, Università Cattolica del Sacro Cuore, 20123 Milan, Italy; 4 Department of Pediatric Neurology and Psychiatry, V. Buzzi Children’s Hospital, ASST Fatebenefratelli Sacco, Via Castelvetro 32, 20154 Milan, Italy

**Keywords:** preterm birth, socio-emotional development, early intervention, longitudinal study, internalizing problems, Bayley Scales, Child Behavior Checklist

## Abstract

Background: Preterm birth increases the risk of socio-emotional difficulties and later behavioral problems. Early identification is essential, but the predictive value of socio-emotional assessments at different ages remains uncertain. Aim: This study sought to examine whether socio-emotional skills at 1, 2, and 3 years predict behavioral outcomes at 4 years in very preterm children. Methods: Fifty-seven preterm children were assessed longitudinally with the Bayley-III Socio-Emotional scale at 1, 2, and 3 years, and with the CBCL 1.5–5 at 4 years. Analyses included correlations, repeated-measures ANOVA, and regression models. Results: Mean socio-emotional scores were within the normative range at all ages, with a modest increase by age 3. Associations were observed between socio-emotional skills at 1 year and behavioral outcomes at 4 years, particularly internalizing and total problems. These associations were weaker at 2 years and not evident at 3 years. Regression analyses indicated that only 1-year socio-emotional scores were significantly associated with later outcomes, although models were unadjusted. Conclusions: Socio-emotional competencies at 1 year were associated with later behavioral outcomes in this sample of very preterm children. These findings suggest that early assessments may contribute to identifying children who could benefit from closer developmental monitoring, although further research with adjusted models is needed.

## 1. Introduction

Preterm birth, a complex and multifactorial event, poses significant challenges to child development, extending far beyond the neonatal period [1]. Advances in neonatal care have dramatically improved survival rates, shifting clinical and research focus towards optimizing long-term neurodevelopmental and psychological outcomes [2,3]. Among the areas of vulnerability for preterm infants is socio-emotional development, encompassing emotional regulation, interpersonal skills, and behavioral management, often in the absence of major cognitive or neurosensory deficits [4,5].

Difficulties in this domain can emerge in the first years of life and, if not adequately identified, may consolidate into stable patterns of functioning, increasing the risk for behavioral and emotional disorders later in childhood [6,7]. Research indicates that up to a quarter of very preterm infants may exhibit delays or deficits in socio-emotional functioning, underscoring the urgency for enhanced clinical vigilance and preventive strategies [8].

The early assessment of socio-emotional competencies is therefore essential. Standardized tools like the Bayley Scales of Infant and Toddler Development (Bayley-III) include a socio-emotional scale designed to identify early signs of atypical development [9]. However, the longitudinal predictive power of these early assessments for specific behavioral outcomes in the preterm population requires further elucidation. While some studies have documented associations, the specific age at which assessment is most predictive remains a critical question for designing efficient early screening protocols [10,11].

This study aims to fill this gap by investigating the longitudinal trajectory of socio-emotional skills in preterm children from 1 to 3 years of age and evaluating their predictive value for emotional and behavioral outcomes at 4 years, as measured by the Child Behavior Checklist (CBCL) [12]. We hypothesized that lower socio-emotional scores on the Bayley-III in the first year of life would be associated with higher rates of behavioral and emotional problems at preschool age, and that this predictive power would be expected to be stronger in the earliest assessment.

## 2. Materials and Methods

### 2.1. Study Context and Ethical Approval

The present study is part of a broader longitudinal research project that started in 2013 in collaboration with CRIdee (Department of Psychology, Catholic University of the Sacred Heart, Milan, Italy) and the Neonatal Intensive Care Unit (NICU) and Obstetric Department of the Buzzi Children’s Hospital in Milan (Milan, Italy). The research follows very preterm infants (e.g., <32 wks’ga) from birth to 7 years of age. The aim of this broad study was to investigate, from a longitudinal perspective, the impact of preterm birth on child development while dedicating particular attention to the parental couple and to the effects and consequences that this event has on the triad.

The study was conducted following the ethical principles outlined in the Declaration of Helsinki and with the standards of good clinical practice. The research protocol was reviewed and approved by the Ethics Committee of Azienda Ospedaliera, Istituti Clinici di Perfezionamento, Milano (protocol number: 1171, 11 December 2012). All participants were fully informed about the aims and procedures of the study and provided written informed consent before participation. Data were collected and stored in anonymized form to ensure confidentiality and privacy. Participation was entirely voluntary, and individuals were free to withdraw from the study at any time without any consequences for the psychological or medical support they were receiving.

### 2.2. Participants

The initial cohort comprised very preterm infants recruited within an ongoing longitudinal study that began in 2012 and included children born from 2008 onward. By January 2026, a total of 915 children had been recruited into the broader study. Of these, only 57 children completed all four assessment time points (1, 2, 3, and 4 years) and were therefore included in the present analyses. Attrition was primarily due to missed follow-up visits or incomplete participation across time points. Detailed information regarding reasons for dropout or missed visits was not available due to privacy regulations. Differences between included participants and those lost to follow-up were not formally tested and represent a potential source of selection bias. Children were included in the study at birth based on two clinical criteria: a gestational age of less than 31 weeks and 6 days (31 + 6 weeks) or a birth weight of less than 1500 g. All participants were enrolled at birth and monitored longitudinally through to school age using a series of standardized assessments administered at predetermined developmental stages. This protocol was designed to track neuropsychological development in the early years of life.

The initial cohort comprised all very preterm infants admitted to the NICU between 2013 and 2021 who met inclusion criteria (gestational age < 32 weeks and/or birth weight < 1500 g). Of these, a larger number were enrolled at birth; however, only children with complete data at all four time points (1, 2, 3, and 4 years) were included in the present analyses (final N = 57). Attrition was primarily due to missed follow-up visits or incomplete assessments. Differences between included participants and those lost to follow-up were not formally tested and represent a potential source of selection bias. The final sample included 32 males (56%) and 25 females (44%). None of the neonates enrolled in the had major lesions (e.g., severe IVH or PVL) at brain MRI performed at 40 weeks post-menstrual age. Infants with major brain injury, defined as intraventricular hemorrhage grade > II and/or periventricular leukomalacia (PVL), were excluded. Key neonatal characteristics are summarized in Table 1.

The mean gestational age was 28.8 weeks (SD = 2.53; range: 23–31 weeks), and the mean birth weight was 1109.9 g (SD = 373.2; range: 491–2450 g). Although the inclusion criteria specified birth weight <1500 g, some infants with higher birth weight (up to 2450 g) were included due to gestational age <32 weeks, consistent with NICU follow-up criteria. This explains the observed upper birth-weight range. The mean length of neonatal hospital stay was 61.92 days (SD = 26.34; range: 15–128 days), reflecting the initial clinical severity common in this population. Regarding plurality, 25% of the sample were monochorionic twins, 14% were dichorionic twins, and 5% were triplets. The mode of delivery was spontaneous vaginal birth in 13% of cases, emergency cesarean section in 57%, and elective cesarean section in 30%.

The Apgar score, a standard measure of neonatal well-being, was used to assess the clinical condition at birth. The mean Apgar score at 1 min was 5.52 (SD = 1.42), indicating moderate initial distress. By 5 min, the mean score had improved to 7.69 (SD = 1.13), suggesting adequate recovery for most infants, although some continued to present with scores below the optimal range.

### 2.3. Measures

In the present study, early socio-emotional development and later emotional-behavioral outcomes were assessed using standardized psychometric instruments.

#### 2.3.1. Socio-Emotional Development

Bayley Scales of Infant and Toddler Development—Third Edition (Bayley-III): The Bayley Scales of Infant and Toddler Development—Third Edition (Bayley-III) [9] is a widely used standardized instrument for analyzing child development between 1 and 42 months of age, with particular attention to the preterm population. The Bayley-III comprises five distinct scales that assess fundamental areas of development: Cognitive, Language, Motor, Socio-Emotional (SE), and Adaptive Behavior (GAC). The Cognitive, Language, and Motor scales are directly administered to the child, while the Socio-Emotional and Adaptive Behavior scales are caregiver-completed questionnaires.

For this study, only the scores from the Socio-Emotional (SE) scale were considered. This parent-report questionnaire evaluates the achievement of important developmental milestones, including the child’s ability to interact and utilize a range of emotions, experiences, and expressions, as well as to understand various emotional signals and process feelings through words and other symbols. It includes items assessing the domain of emotional-functional ability (e.g., self-regulation, interest in the world), the need for communication, the interactive use of emotions, and the use of emotional signals or gestures to solve problems. Each of the Bayley-III scales provides a composite index score (Mean = 100, SD = 15), while each subscale provides a norm-referenced scaled score (Mean = 10, SD = 3). Prior validation studies report strong internal consistency for the overall Bayley-III scales (e.g., α values ranging from 0.86 to 0.91 for cognitive, language, and motor domains in localized samples) [13]. Cronbach’s alpha for the Bayley-III socio-emotional scale was 0.809 in the present sample.

#### 2.3.2. Behavioral and Emotional Outcomes

Child Behavior Checklist for Ages 1.5–5 (CBCL 1.5–5): To assess behavioral and emotional outcomes, parents of children aged 4 years completed the Child Behavior Checklist for Ages 1.5–5 (CBCL 1.5–5) [12]. This is a standardized parent-report instrument designed for the early identification of emotional and behavioral difficulties in children aged 18 months to 5 years, representing a revision of the earlier CBCL 4–18 specifically adapted for this age range. It consists of 99 items exploring various dimensions of child behavior. Parents are asked to rate each item based on how accurately it describes their child’s behavior over the preceding two months. Responses are provided on a three-point scale: 0 = not true, 1 = partly true or sometimes true, and 2 = very true or often true. The scoring procedure for the CBCL 1.5–5 yields three primary profiles of behavioral and emotional functioning: Internalizing Problems, Externalizing Problems, and Total Problems. The CBCL 1½–5 has consistently shown excellent internal consistency in recent evaluations—Cronbach’s α is typically reported as satisfactory in both community and clinical samples (e.g., α ≈ 0.95) [10]. Cronbach’s alpha for the CBCL total scale was 0.767 in the present sample.

### 2.4. Statistical Analysis

Data were analyzed using SPSS version 29. Descriptive statistics (means, standard deviations) were computed at each time point. Age was corrected for prematurity at all assessment points.

Pearson correlations were calculated between Bayley-III socio-emotional scores (1, 2, and 3 years) and CBCL scores at 4 years. To reduce the risk of Type I error, results were interpreted cautiously in light of multiple testing. No formal correction for multiple comparisons was applied, as the analyses were exploratory in nature; this is acknowledged as a limitation.

A repeated-measures ANOVA was conducted to examine longitudinal changes in socio-emotional development. Assumptions of normality and sphericity were tested.

Multiple linear regression analyses were performed, entering socio-emotional scores at 1, 2, and 3 years simultaneously. These models were unadjusted and did not include potential confounders (e.g., gestational age, sex, socioeconomic status), and results should therefore be interpreted as exploratory associations.

Missing data were handled using listwise deletion, resulting in a final sample of 57 children with complete longitudinal data.

No formal statistical comparison was conducted to directly test whether the predictive value of the 1-year assessment differed significantly from the 2- or 3-year assessments; therefore, conclusions regarding relative predictive strength should be interpreted cautiously.

## 3. Results

### 3.1. Descriptive Statistics

The mean socio-emotional scores of the preterm sample were within the normative range at all ages. Table 2 provides an overview of the sample’s socio-emotional development across the first three years of life. Scores showed a significant increase from 1 to 3 years (F(2, 55) = 7.047, *p* = 0.002), with a moderate effect size (partial η^2^ = 0.125).

At 4 years, CBCL 1.5–5 scores showed that the sample was predominantly within the normal range. For Internalizing Problems, 68.4% of the sample was within the normal range, 12.3% in the borderline area. Notably, 19.3% of children fell within the clinical range for internalizing problems, which represents a non-negligible proportion and suggests the presence of meaningful emotional vulnerability in this population. For Externalizing Problems, 89.5% were within the normal range, 7.0% in the borderline area, and 3.5% in the clinical range. Additionally, for Total Problems, 82.5% were within the normal range, 14.0% in the borderline area, and 3.5% in the clinical range. This distribution indicates a slightly higher proportion of children in the borderline and clinical ranges for internalizing problems compared to externalizing problems.

### 3.2. Correlation Analysis

Full correlation coefficients for all CBCL scales are reported in Table 3. Given the number of comparisons, results should be interpreted cautiously. At 1 year, strong negative correlations were found with 8 of the 11 CBCL scales, including Total Problems (r = −0.388, *p* < 0.01), Internalizing Problems (r = −0.383, *p* < 0.01), and Externalizing Problems (r = −0.348, *p* < 0.01). At 2 years, significant correlations persisted for only 4 scales (e.g., Aggressive Behavior, Total Problems). By 3 years, only the correlation with the Withdrawn scale remained significant (r = −0.325, *p* = 0.014). Given the number of comparisons, no correction was applied, and findings should be interpreted cautiously.

### 3.3. Regression Analysis

Multiple regression models confirmed the unique predictive value of the 1-year assessment (Table 4). For Internalizing Problems, the overall model was significant, F(3, 53) = 3.136, *p* = 0.033, and explained 15.1% of the variance (Adjusted R^2^ = 0.103). The 1-year socio-emotional score was a significant negative predictor (β = −0.446, *p* = 0.036). For Externalizing Problems, the overall model was also significant, F(3, 53) = 3.123, *p* = 0.033, and explained 15.0% of the variance (Adjusted R^2^ = 0.102). However, none of the individual predictors reached statistical significance (*p* > 0.05). Finally, for Total Problems, the overall model was significant, F(3, 53) = 3.372, *p* = 0.025, and explained 16.0% of the variance (Adjusted R^2^ = 0.113). Only the 1-year socio-emotional score showed a significant negative association (β = −0.433, *p* = 0.041). Scores at 2 and 3 years did not add significant predictive power to any of the models. In all models, assumptions of multicollinearity were met, with tolerance values ranging from 0.372 to 0.943 and VIF values from 1.639 to 2.691, indicating no relevant issues.

## 4. Discussion

This longitudinal study aimed to delineate the predictive power of early socio-emotional skills on later emotional-behavioral outcomes in a cohort of very preterm children without severe brain damage (no IVH > II and or PVL). Our central finding is a moderate negative association between socio-emotional competencies assessed at 1 year of age and subsequent internalizing and total problems at preschool age, with this predictive power significantly attenuating by ages 2 and 3.

The descriptive statistics indicated that the preterm children, as a group, generally showed socio-emotional development within the normative range. By the third year, a slight positive increase was observed relative to the normative mean. This pattern is particularly noteworthy given that existing literature often highlights socio-emotional development as an area of vulnerability for preterm infants, with atypical patterns emerging early in life and up to a quarter of this population potentially experiencing delays [6,7,8]. The fact that our sample demonstrated normative or even slightly superior average scores suggests that, despite their intrinsic vulnerability, these children did not exhibit significant socio-emotional delays as a group. This positive trajectory could be attributed to the effectiveness of modern neonatal care [14], the presence of unmeasured protective factors such as the quality of parent–child interactions [15], or a phenomenon of “catch-up growth” and skill consolidation fostered by a supportive care environment [16]. Regarding emotional-behavioral functioning, assessed at 4 years, the sample fell within the normal range, although there was a slight tendency towards higher scores in the internalizing dimensions compared to externalizing ones. This subtle yet persistent vulnerability in the internal sphere aligns with previous findings showing that preterm children are particularly prone to anxiety, withdrawal, and emotional dysregulation [17,18]. Stressful early experiences, such as those associated with the NICU stay, have been linked to alterations in the hypothalamic–pituitary–adrenal (HPA) axis, a biological system central to stress regulation, and these alterations may underlie the emergence of internalizing difficulties. Painful procedures in the NICU have also been associated with subsequent internalizing problems. Thus, the persistence of even a slight vulnerability underscores the importance of long-term monitoring. While the group as a whole did not present clinically significant difficulties, these findings highlight the heterogeneity of developmental outcomes and the possibility that protective factors mitigated the expected risks. The correlation analyses revealed a clear age-dependent pattern, with the strongest associations observed at 1 year; however, these findings should be interpreted cautiously given the exploratory nature of the analyses and the absence of correction for multiple comparisons. During this period, socio-emotional competencies were consistently associated with fewer difficulties across multiple behavioral domains at preschool age. This supports the hypothesis that strong socio-emotional skills in the first year may be associated with lower levels of later difficulties. By the second year, the associations were fewer and weaker, and by the third year, only a single domain showed a significant link. This progressive reduction in predictive capacity emphasizes the importance of very early assessments, through a well-structured follow-up service, suggesting that socio-emotional trajectories may be most strongly shaped during the first year of life through interaction with caregivers and the environment. These findings resonate with prior research underscoring the particular vulnerability of very preterm children and the critical importance of early monitoring and intervention [4,19]. The longitudinal analyses further demonstrated that socio-emotional skills improved progressively between 1 and 3 years of age, suggesting that development is dynamic rather than static. This pattern aligns with transactional models of development in which biological maturation and relational environments interact to promote socio-emotional growth. Clinically, these findings suggest that preterm children are capable of a “catch-up” trajectory, particularly when embedded in sensitive caregiving contexts, but that this trajectory may remain fragile and susceptible to disruption in the presence of environmental stressors. Regression analyses confirmed that only the 1-year assessment predicted later internalizing and total problems, whereas assessments at 2 and 3 years did not add significant predictive power. This result suggests that the first year may show stronger associations with later outcomes in this sample. The predictive power was concentrated on internalizing rather than externalizing problems, which is consistent with meta-analytic evidence showing that preterm children are particularly vulnerable in the domain of emotional regulation rather than overt behavioral difficulties [17,20]. In addition, human studies suggest that early exposure to elevated maternal cortisol may influence brain development, particularly affecting the morphology of the amygdala and hippocampus, and has been associated with later affective symptoms. These findings provide a potential neurobiological pathway linking early stress exposure to internalizing difficulties.

Taken together, the integrated data from descriptive, correlational, longitudinal, and regression analyses provide a coherent picture: although preterm birth is associated with heightened socio-emotional risk, the first year of life appears to show the strongest associations in this sample, suggesting potential avenues for early identification and prevention. These findings may inform future clinical approaches, including a shift from the traditional focus on cognitive and motor milestones towards the integration of standardized socio-emotional screening into follow-up programs before 12 months of corrected age. Such proactive screening would allow for timely interventions targeting the caregiver-infant relationship, which is central to socio-emotional development, and would empower parents with a clearer understanding of their child’s needs, reducing stress and enhancing their sense of competence [21].

It should also be noted that no formal statistical comparison between predictors across ages was conducted; therefore, the interpretation that the 1-year assessment shows stronger associations is based on observed patterns rather than direct statistical testing.

### Limitations and Future Directions

This study has several limitations. Firstly, the relatively small sample size and attrition over time may introduce selection bias and limit generalizability. In particular, only a small proportion of the initially recruited cohort completed all assessment time points, which may increase the risk of selection bias. Secondly, the analyses involved multiple comparisons without formal correction, increasing the risk of Type I error. Thirdly, regression models were unadjusted and did not account for important confounders such as socioeconomic status, parental mental health, or neonatal complications. Fourthly, the reliance on parent-report measures for both predictors and outcomes raises the possibility of shared-method variance.

Additionally, the sample included a high proportion of multiple births (approximately 45%), which was not accounted for in the analyses due to limited statistical power. This may have influenced developmental outcomes.

Finally, psychosocial variables related to the mother–infant relationship (e.g., early intersubjectivity, attachment quality) were not assessed and should be included in future research to better understand developmental pathways.

## 5. Conclusions

This longitudinal study provides preliminary evidence of an association between early socio-emotional competencies and later emotional and behavioral outcomes in very preterm children. The findings suggest that socio-emotional skills assessed at 1 year may show stronger associations with later outcomes compared to assessments at later ages, although this should be interpreted cautiously given the exploratory nature of the analyses and the absence of adjustment for confounding factors.

Despite the inherent vulnerability of preterm infants, our sample displayed average socio-emotional scores generally within the normative range at 1 and 2 years, even showing a slight positive increase by age 3. This encouraging trend may reflect the positive impact of modern neonatal care and dedicated follow-up programs. However, the observed slight elevation in internalizing problems at age 4 aligns with existing literature suggesting a specific emotional fragility in this population, potentially linked to early stress and altered HPA axis function. This underscores the persistence of subtle vulnerabilities that warrant specific attention, even in a context of overall adaptive development. These results may have implications for clinical practice, although these should be interpreted cautiously in follow-up neonatal care. They may support the integration of standardized socio-emotional screening into routine follow-up programs, specifically before 12 months of corrected age. Identifying vulnerabilities at this ultra-early stage, rather than awaiting the manifestation of behavioral problems at 3 or 4 years, offers an opportunity for earlier monitoring and supportive interventions. This proactive approach moves beyond a “wait-and-see” model, enabling interventions to focus on strengthening the primary caregiver-infant relationship, which is a cornerstone of socio-emotional development. Furthermore, early identification can provide parents of preterm infants with greater clarity, reducing their anxiety and empowering them with a clear understanding of their child’s needs and a concrete path of support, thereby fostering parental autonomy, a crucial protective factor for the child’s development.

In conclusion, this longitudinal study found that socio-emotional competencies assessed at 1 year were more strongly associated with behavioral outcomes at 4 years than those assessed at later ages. However, these findings should be interpreted cautiously, given the exploratory nature of the analyses and the absence of adjustment for confounding factors. While early socio-emotional assessment may contribute to developmental monitoring, further research is needed to confirm these findings and to determine their clinical utility.

## Figures and Tables

**Table 1:** Neonatal and demographic characteristics of the preterm sample (N = 57).

Characteristic	Value
Sex, n (%)	
Male	32 (56.1%)
Female	25 (43.9%)
Gestational Age (weeks)	
Mean (SD)	28.8 (2.53)
Range	23–31
Birth Weight (grams)	
Mean (SD)	1109.9 (373.2)
Range	491–2450
Apgar Score, Mean (SD)	
1 min	5.52 (1.42)
5 min	7.69 (1.13)
Length of Stay (days)	
Mean (SD)	61.92 (26.34)
Range	15–128
Plurality, n (%)	
Monochorionic Twins	14 (24.6%)
Dichorionic Twins	8 (14.0%)
Triplets	3 (5.3%)
Mode of Delivery, n (%)	
Spontaneous Vaginal Birth	7 (12.3%)
Emergency Cesarean Section	32 (56.1%)
Elective Cesarean Section	17 (29.8%)

**Table 2:** Descriptive statistics for Bayley-III Socio-Emotional Composite Scores.

Age	Mean	Standard Deviation	Range
1 Year	98.95	13.35	70–140
2 Years	99.75	15.34	51–140
3 Years	105.26	15.31	70–140

**Table 3:** Pearson correlations between Bayley-III socio-emotional scores and CBCL scores at 4 years.

	1 Year (r)	*p*	2 Years (r)	*p*	3 Years (r)	*p*
Emotional Reactivity	−0.302	0.022	−0.180	0.182	−0.102	0.446
Anxious/Depressed	−0.355	0.007	−0.201	0.133	−0.085	0.524
Withdrawn	−0.318	0.016	−0.210	0.117	−0.325	0.014
Somatic Complaints	−0.220	0.102	−0.145	0.283	−0.090	0.507
Attention Problems	−0.240	0.073	−0.198	0.139	−0.110	0.418
Aggressive Behavior	−0.351	0.008	−0.318	0.017	−0.145	0.279
Other Problems	−0.383	0.003	−0.304	0.022	−0.120	0.372
Internalizing Problems	−0.383	0.003	−0.210	0.116	−0.098	0.468
Externalizing Problems	−0.348	0.008	−0.320	0.016	−0.132	0.326
Total Problems	−0.388	0.003	−0.300	0.024	−0.125	0.350

Note: Negative correlations indicate that higher socio-emotional scores are associated with fewer behavioral problems. No formal correction for multiple comparisons was applied; results should therefore be interpreted cautiously.

**Table 4:** Multiple linear regression models predicting CBCL scores at 4 years.

Outcome	Predictor	β	*p*	R^2^	Adj. R^2^	F (df)	*p* (Model)
Internalizing	1 year	−0.446	0.036	0.151	0.103	3.136 (3, 53)	0.033
	2 years	0.096	0.612				
	3 years	−0.012	0.943				
Externalizing	1 year	−0.347	0.100	0.150	0.102	3.123 (3, 53)	0.033
	2 years	−0.155	0.412				
	3 years	0.184	0.262				
Total Problems	1 year	−0.433	0.041	0.160	0.113	3.372 (3, 53)	0.025
	2 years	−0.044	0.816				
	3 years	0.125	0.443				

## Data Availability

The raw data supporting the conclusions of this article will not be made available by the authors due to privacy and ethical restrictions connected to patients’ data.
